# Interleukin-27 Is a Potent Inhibitor of *cis* HIV-1 Replication in Monocyte-Derived Dendritic Cells via a Type I Interferon-Independent Pathway

**DOI:** 10.1371/journal.pone.0059194

**Published:** 2013-03-20

**Authors:** Qian Chen, Sanjay Swaminathan, De Yang, Lue Dai, Hongyan Sui, Jun Yang, Ronald L. Hornung, Yanmei Wang, Da Wei Huang, Xiaojun Hu, Richard A. Lempicki, Tomozumi Imamichi

**Affiliations:** 1 Laboratory of Human Retrovirology, Applied and Developmental Research Directorate (ADD), Science Application International Corporation (SAIC)-Frederick, Inc., Frederick National Laboratory for Cancer Research, Frederick, Maryland, United States of America; 2 Laboratory of Molecular Immunoregulation, Basic Research Program Directorate, SAIC-Frederick, Inc., Frederick National Laboratory for Cancer Research, Frederick, Maryland, United States of America; 3 Laboratory of Immunopathogenesis and Bioinformatics, ADD, SAIC-Frederick, Inc., Frederick National Laboratory for Cancer Research, Frederick, Maryland, United States of America; 4 Immunological Monitoring Laboratory, ADD, SAIC-Frederick, Inc., Frederick National Laboratory for Cancer Research, Frederick, Maryland, United States of America; University Hospital Zurich, Switzerland

## Abstract

IL-27, a member of the IL-12 family of cytokines, plays an important and diverse role in the function of the immune system. Whilst generally recognized as an anti-inflammatory cytokine, in addition IL-27 has been found to have broad anti-viral effects. Recently, IL-27 has been shown to be a potent inhibitor of HIV-1 infection in CD4+ T cells and macrophages. The main objective of this study was to see whether IL-27 has a similar inhibitory effect on HIV-1 replication in dendritic cells (DCs). Monocytes were differentiated into immature DCs (iDCs) and mature DCs (mDCs) with standard techniques using a combination of GM-CSF, IL-4 and LPS. Following differentiation, iDCs were infected with HIV-1 and co-cultured in the presence or absence of IL-27. IL-27 treated DCs were shown to be highly potent inhibitors of *cis* HIV-1, particularly of CCR5 tropic strains. Of note, other IL-12 family members (IL-12, IL-23 and IL-35) had no effect on HIV-1 replication. Microarray studies of IL-27 treated DCs showed no up-regulation of Type I (IFN) gene expression. Neutralization of the Type-I IFN receptor had no impact on the HIV inhibition. Lastly, IL-27 mediated inhibition was shown to act post-viral entry and prior to completion of reverse transcription. These results show for the first time that IL-27 is a potent inhibitor of *cis* HIV-1 infection in DCs by a Type I IFN independent mechanism. IL-27 has previously been reported to inhibit HIV-1 replication in CD4+ T cells and macrophages, thus taken together, this cytokine is a potent anti-HIV agent against all major cell types targeted by the HIV-1 virus and may have a therapeutic role in the future.

## Introduction

IL-27, a member of the IL-12 family of cytokines [1], has been shown to play important roles in both the innate and adaptive immune systems. The IL-12 family (IL-23, IL-27 and IL-35) is made of various combinations of shared α-chains and β-chains [2] and IL-27 is made up of two subunits, namely the IL-27p28 subunit and an Epstein-Barr virus induced gene 3 (EBI3) subunit [3]. The cytokine interacts with the IL-27 receptor, a heterodimer of IL-27Rα (WSX-1) and glycoprotein 130 (gp130) [4] and signaling occurs via the Janus kinase (JAK)/signal transducer and activator of transcription (STAT) pathway. Typically STATs 1 and 3 are activated following engagement of IL-27 with its receptor [5]. The first description of the function of IL-27 demonstrated that it could lead to proliferation of naïve T cells with subsequent differentiation into T helper 1 cells [3], [6], and hence IL-27 was initially thought of as a pro-inflammatory cytokine. However, due to its ability to affect the function of multiple T cell subsets such as Tr1 cells, T follicular cells (Tfh), Th17 cells and T regulatory cells (Tregs) and ability to modulate the production of anti-inflammatory cytokines such as IL-10, IL-27 is now thought to be predominantly an anti-inflammatory cytokine [7].

HIV-1 infection is an important viral pathogen infecting more than 34 million people worldwide. It cripples the adaptive immune system by targeting CD4+ T cells, but can also infect other cells such as macrophages, dendritic cells (DCs) and tissues of the central nervous system. Current therapeutic strategies employ using multiple drugs targeting different aspects of the viral replication pathway. Whilst highly effective, they do have limitations including adverse effects and the need for lifelong treatment due to the inability to purge the virus from latent reservoirs. Novel therapeutic strategies to treat HIV-1 have emerged and in the last few years, a number of reports have shown that IL-27 has potent anti-HIV-1 properties *in-vitro* [8], [9], [10]. Recombinant IL-27 administered to both CD4+ T cells and monocyte derived macrophages (MDMs) were shown to potently inhibit HIV-1 replication [8], [9]. Further experiments established that IL-27 mediated HIV-1 inhibition was occurring via the induction of interferon like genes but not by Type I interferons directly [10].

Dendritic cells (DCs) are a group of professional antigen presenting cells which play vital roles in initiating the adaptive immune response and in tolerance [11]. They present peptide antigens via HLA molecules to T cells in lymph nodes and provide activating stimuli to these cells [12]. They have been divided into three types: classical DCs, plasmacytoid DCs found in the blood and Langerhans cells (LCs) which are found in tissue (skin and mucosal surfaces) [13]. Immature DCs (iDCs) line mucosal surfaces where they constantly survey the environment for pathogens. Recognition of pathogens by iDCs leads to migration to lymph nodes, up-regulation of activation markers and high efficiency presentation of antigen to T cells, features which define mature DCs (mDCs). DCs have important roles in HIV-1 infection in terms of disseminating the virus to T cells and also in initiating adaptive immune responses against the virus. DCs can be infected with HIV-1 in two distinct ways: *trans*-infection can occur via infectious synapses or via exocytosis of HIV-associated exosomes [14] and *cis*-infection following de novo viral production in DCs [15]. Strategies to reduce HIV-1 infection in DCs may be valuable in limiting the catastrophic lymphoid destruction seen early in the disease course [16], [17], [18].

In the current study, the aim was to investigate the effects of IL-27 on *cis* HIV-1 infection in DCs. We clearly demonstrate that iDCs and mDCs highly express the IL-27 receptor and engagement of IL-27 with its receptor leads to rapid phosphorylation of STAT-1, -3 and -5. IL-27 is shown for the first time to be a potent inhibitor of *cis* HIV-1 replication in both iDCs and mDCs with a CCR5-tropic virus. With regard to the mechanism, microarray profiling of DCs exposed to IL-27 demonstrate that the anti-HIV effect of IL-27 is occurring in an interferon (IFN) independent manner. Lastly, IL-27 mediated inhibition of HIV-1 in iDCs appears to work post viral entry and prior to completion of reverse transcription.

## Materials and Methods

### Ethics Statement

Ethics approval for these studies was granted by the institutional review board of NIH.

### Generation of DC Subtypes

CD14+ monocytes were isolated from healthy donor peripheral blood enriched leukopacks (Blood Bank, National Institute of Health, Bethesda, MD) using MACS CD14 MicroBeads (Miltenyi Biotec, Auburn, CA, USA). Monocyte derived immature DCs (iDCs) were generated by incubating 0.5×10^6^/ml monocytes in G4 medium (RPMI 1640 containing 10% fetal bovine serum (Hyclone, UT, USA), 2 mM glutamine, 25 mM HEPES [N-2-hydroxyethylpiperazine-N′-2-ethanesulfonic acid] (Quality Biological, Gaithersburg, MD, USA), 10 µg/ml gentamicin, 50 ng/mL GM-CSF (R&D Systems, Minneapolis, MN, USA) and 50 ng/mL IL-4 (R&D Systems) at 37°C in a CO2 (5%) incubator for 7 days. The culture media was changed with fresh G4 media every 3–4 days. The mature DCs (mDCs) were induced from the iDCs by stimulation with 1 µg/ml lipopolysaccharide (LPS) (Sigma-Aldrich, St Louis, MO, USA) for two more days. The Langerhans like cells (LLCs) were generated by incubating monocytes in G4 medium containing 5 ng/ml of TGF-β1 (R&D systems) [19]. The cellular phenotype was confirmed by Fluorescence-activated cell sorting (FACS) as previously described [20]. Cell viability was assessed by trypan blue exclusion method and counted using the Invitrogen Countess Automated Cell counter (Carlsbad, CA, USA).

### Reagents and Virus Stock preparation

Recombinant human IL-12, IL-23, IL-27, IFN-α, anti-IFN-α receptor chain 2 neutralizing antibody, and isotype control antibody were purchased from R&D Systems. Recombinant IL-35 was purchased from Enzo (New York, NY, USA). Plasmids, pNL4.3 encoding full length of CXCR4 tropic HIV-1 strain, HIV-1_NL4.3,_ [21], pNL4-3.Luc.R^−^.E^−^ [22], [23] and pEFH-VSVG, encoding vesicular stomatitis virus G envelope glycoprotein [24] were obtained from the NIH AIDS reagent program. The infectious HIV-1_NL4.3_ was prepared by transfection of pNL4.3 to RD cells (ATCC, Manassas, VA, USA) as previously described [25]. The CCR5 tropic HIV-1 strain, HIV-1_Ba-L_, was obtained from Advanced Biotechnologies Inc (Columbia, MD, USA), and the virus infectious titer (50% tissue culture infective dose, TCID_50_) was determined as previously described [26]. A pseudo typed virus was prepared by co-transfection of both pNL4-3.Luc.R^−^.E^−^ and pEFH-VSVG into 293T cells (ATCC) as previously described [27], and virus amounts were quantitated by the p24 antigen capture kit (Perkin-Elmer, Shelton, Connecticut, USA).

### HIV-1 Infection and Replication Assay

The iDCs, mDCs or LLCs (1×10^6^ cells) were infected with 1000 TCID_50_ HIV-1_NL4.3_ or HIV-1_Ba-L_ for 2 hours at 37°C and then washed 3 times in RPMI-1640. The infected cells were cultured at 0.5×10^6^ cells/ml in the presence or absence of IL-27 (in G4 medium) for 14 days. Half of the culture medium was changed with fresh G4 medium with the same concentrations of IL-27 every 3 to 4 days. For the experiments where iDCs were pretreated with IL-27, newly differentiated iDCs were exposed to IL-27 at a dose of 100 ng/ml for 48 hours and then washed twice in PBS. They were then infected with HIV-1_Ba-L_ as described above, washed 3 times in RPMI-1640, resuspended in G4 medium without IL-27 and cultured for a further 14 days. HIV-1 replication was determined by measuring p24 antigen in the culture supernatant using a p24 antigen capture assay (Perkin-Elmer).

### HIV-1 Single-round Infection Assay

2×10^6^ iDCs were incubated with 1 ml pseudo typed HIV_NL4-3.Luc.R-.E-_ (1 mg/ml p24) for four hours and then washed three times with RPMI-1640. Cells were re-suspended at 1×10^6^ cells/ml in G4 medium and cultured for 4 days. Cells were lysed and luciferase activity was measured with Bright-Glo luciferase assay system (Promega, Fitchburg, WI, USA). Quantitative analysis of viral cDNA late products was performed as previously described [28]. Copy number estimates of cDNA late product were determined by real-time PCR on a BioRad IQ5 RT-PCR detection system. The cDNA late product copy numbers were normalized by the cell numbers determined by real-time PCR using CCR5-specific primers [8].

### Quantitation of Cytokine Concentration in Culture Supernatants

The iDCs were cultured in the presence or absence of 100 ng/ml of IL-27 for 1, 2, 4 or 7 days, and the concentrations of IFN-α in the culture supernatants was determined using a Human-IFN-α Multi-Subtype ELISA kit (R&D Systems) which could detect 14 out of 15 identified human IFN-α subtypes (IFN-αA, IFN-α2, IFN-αD, IFN-αB2, IFN-αC, IFN-αG, IFN-αH, IFN-αI, IFN-αJ1, IFN-αK, IFN-α1, IFN-α4a, IFN-α4b, and IFN-αWA.). The concentrations of multiple cytokines in the culture supernatants were analyzed using Luminex 100 instrumentation (Luminex, Austin, TX, USA) with Milliplex assay kits (Millipore, Billerica, MA, USA) and analyzed, using a Logistic-5PL regression method, with the Bio-Plex manager 5.0 software (Bio-Rad Laboratories, Hercules, CA USA).

### FACS Analysis

1×10^6^ DCs were blocked with 2% human AB serum for 10 minutes and were stained for 30 minutes at room temperature in the dark in PBS containing 2% (wt/vol) bovine serum albumin (Sigma Aldrich) with the following antibodies: FITC-conjugated anti-CD4 (BD Biosciences, San Jose, CA, USA), FITC-conjugated anti-CCR5 (R&D Systems), PE-conjugated anti-CXCR4 (R&D Systems), PE-conjugated anti-WSX-1 (R&D Systems) or PE-conjugated anti-DC-SIGN (R&D Systems). The iDCs were washed three times in FACS buffer (PBS in 1% wt/vol FBS and 0.02% Sodium Azide) and then analyzed immediately with a FACSCalibur flow cytometer (BD Biosciences). Specificity of staining was determined through the use of proper isotype-matched control antibodies.

### Microarray Analysis

1×10^6^ DCs were stimulated in the presence or absence of 100 ng/ml IL-27 for 48 hours. The iDCs were washed with cold PBS and then RNA was isolated using a Qiagen RNeasy spin column kit (Qiagen, Hilden, Germany) and RNA quantification and quality were assayed using standard procedures. The gene expression profile in IL-27-treated iDCs versus mock treated iDCs was conducted using the human GeneArray ST 1.0 microarray (Affymetrix, Santa Clara, CA, USA). In brief, total RNA was synthesized for 1st and 2nd strands of cDNA, in vitro transcription, the second cycle of cDNA synthesis and fragmentation and terminal labeling, which was then followed by the Affymetrix protocol. In total, six arrays (mock and IL-27-treated samples from 3 donors) were analyzed using Partek Pro (Partek, St. Louis, MO, USA). Differentially expressed genes were selected by a p value ≤0.05 and fold change less than or greater than two fold between IL-27-treated and mock samples. The biologic functions and involved pathways of the selected gene subsets were determined using DAVID, a bioinformatics analysis tool [29]. The microarray data are available at the Gene Expression Ommunibus under accession no. GSE44732.

### Real-time PCR

To quantitate the relative amount of the expression of the IFN genes, real time PCR was performed using the iCycler real-time PCR detection system (Bio-Rad). Taqman probes for IFN genes were obtained from Applied Biosystems (Foster City, CA, USA). The PCR cycling conditions were 50°C for 2 minutes, denaturation at 95°C for 10 minutes, followed by 39 cycles (15 seconds at 95°C, 60 seconds at 60°C). To normalize results, GAPDH was used as the housekeeper gene. The relative expression (ΔCt) and quantification (RQ = 2^−(ΔΔCt)^) for each gene was calculated using the ΔΔCt method, as suggested by the manufacturer.

### Western Blotting to Analyze STAT Activation profile

iDCs (1×10^6^) were stimulated with IL-27 (100 ng/mL) at 37°C for a period of time as specified. At the end of stimulation, the iDCs were washed 3 times with cold PBS. The iDCs were lysed by adding 100 µL of radio-immunoprecipitation assay (RIPA) buffer containing a phosphatase inhibitor cocktail (Thermo Fisher Scientific, Waltham, MA) and protease inhibitor cocktail (Sigma-Aldrich, St. Louis, MO). Protein quantity was estimated using the BCA protein assay reagent (Thermo Fisher Scientific). 20 µg of protein was loaded onto a 4–10% SDS-polyacrylamide gel and subsequently transferred onto a 0.45 µm nitrocellulose membrane. Membranes were probed with phosphorylated and unphosphorylated STAT antibodies (all antibodies were from Cell Signaling Technology, Danvers, MA, USA except for unphosphorylated STAT-2 which was from Santa Cruz Biotechnology, Inc., Dallas, TX, USA). The primary antibody was detected with horseradish peroxidase-conjugated anti-rabbit IgG (eBioscience, San Diego, CA, USA) and signals were detected with the ECL Plus Western blotting detection system (GE-Healthcare, Waukesha, WI, USA) [10].

### Statistical Analysis

Differences in HIV-1 inhibition and cytokine production between the untreated and IL-27- treated cells were calculated by the student t test using the StarView program (Abacus Concepts, (Berkeley, CA, USA). P values <0.05 were considered statistically significant.

## Results

### Monocyte Derived iDCs and mDCs Express the IL-27 Receptor

**Figure 1:**
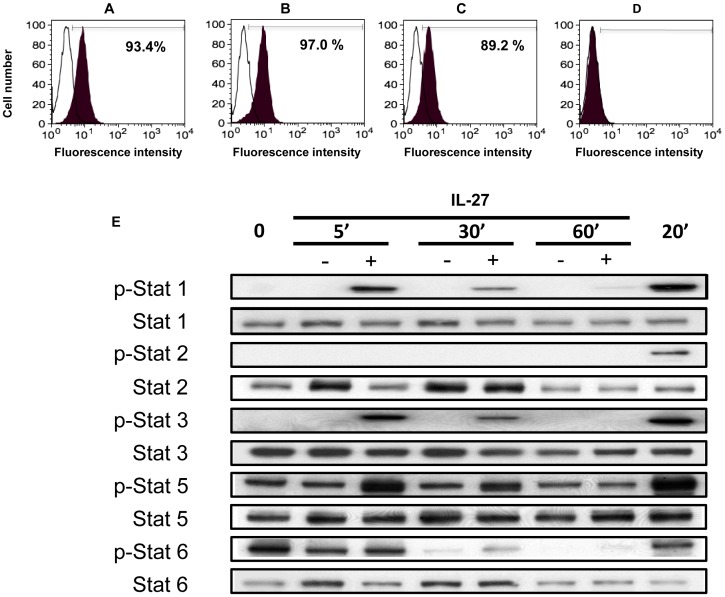
Expression levels of IL-27Rα (WSX-1) in DCs and STAT activation following IL-27 treatment. A–D: The expression level of the IL-27 receptor (WSX-1) was measured using flow cytometry on the following cells: A, Freshly isolated monocytes B, iDCs C, mDCs D, LLC. These cells were stained with either a PE-conjugated anti-WSX-1 (black) antibody or an isotype control (white). Data indicates a representative result from a single donor from three independent experiments. The numbers in the figures show percentage of positive cells, and the Mean Fluorescence Intensity (MFI) of monocytes, iDCs and mDCs were 8.75, 9.13 and 6.3, respectively. E: Western blotting was used to measure the phosphorylation status of STAT-1, -2, -3, -5, and -6 in IL-27 or untreated iDCs at a number of time points. For each phosphorylated STAT measured, the unphosphorylated protein was also measured to show that the profiles were not due to loading differences.

To understand the possible functional relevance of IL-27 in DCs, the first step was to determine whether IL-27Rα (WSX-1) was expressed in these cells. Using flow cytometry, 93% of monocytes, 97% of iDCs and 89% of mDCs were found to express the IL-27Rα, while LLCs- completely lacked this subunit of the receptor (Figure 1A–D). This was suggestive that iDCs and mDCs would be responsive to IL-27 and that LLCs would not respond to IL-27. To confirm that iDCs could respond to IL-27, a time-course experiment was performed investigating the activation of STATs after culturing iDCs in the presence or absence of 100 ng/ml of IL-27. It was observed that phosphorylated STAT-1, -3 and -5 were markedly up-regulated after five minutes and the levels of these proteins declined over the first hour (see Figure 1E). STAT-2 and -6 were not significantly altered in IL-27 treated iDCs compared to mock treated cells. It should be noted that as IL-4 was in the G4 culture medium, STAT-5 and -6 activation was expected at the initial time points (IL-4 activates STAT-5 and -6). However it is clear that STAT-5 activation occurs to an even greater extent in the presence of IL-27 at the early time points (5 and 30 minutes). The alteration in STAT profiles cannot be simply explained by differences in viability of cells. In untreated iDCs the viability was 90.7±3.8% and in IL-27 treated iDCs it was 92.0±1.4% (p>0.05). Previously, human monocyte derived DCs have been shown to activate STAT-1 [30] but this is the first description of STAT-3 and -5 also being activated in human monocyte derived DCs following engagement of IL-27 with its receptor. For completeness, further experiments looked at surface expression of known DC maturation markers (CD83, CD11c, CD80, CD86, HLA-ABC, HLA-DR, CD58, CCR7 and CCR5) in iDCs exposed to IL-27 for two days and compared these to iDCs exposed to LPS for two days (see Figure S1). This showed that IL-27 was capable of up-regulating CD11c, HLA-ABC and HLA-DR to a similar extent to DCs exposed to LPS, but was not able to induce the expression of CD80, CD83 or CD86 to the same levels as seen with LPS.

### iDCs and mDCs Express HIV-1 Receptors and can be Infected with HIV-1

**Figure 2:**
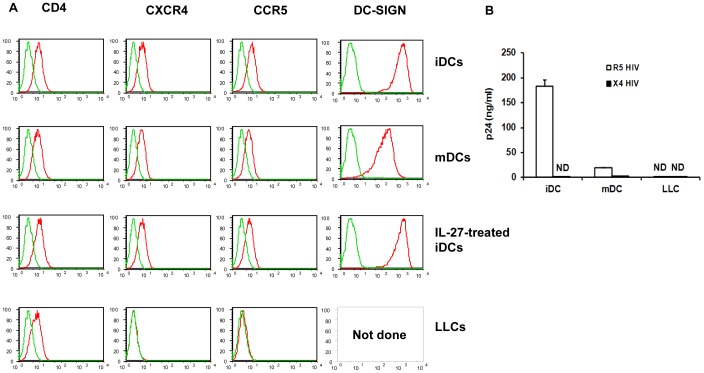
HIV-1 co-receptor expression in DCs and HIV-1 infection rates in DC subsets. A: The expression level of CD4, CXCR4 and CCR5 was measured using flow cytometry in iDCs, mDCs, LLC and IL-27-treated iDCs. DC-SIGN was also measured but only in iDCs, mDCs and IL-27 treated iDCs. The red color in the plots indicates specific antibody staining whilst green represents the isotype control. B: iDCs, mDCs and LLCs were infected with either HIV-1_Ba-L_ or HIV-1_NL4.3_ as described in the Materials and Methods. HIV-1 replication was measured using HIV-1 p24 antigen capture kit as described in the Materials and Methods. These graphs are representative data from a single donor from three independent experiments.

The next goal was to investigate expression levels of HIV (co)-receptors CD4, CCR5 and CXCR4 in each of the DC subtypes that were generated (iDCs, IL-27 treated iDCs, mDCs and LLCs). Flow cytometry clearly demonstrated that iDCs and mDCs expressed all three receptors on their cell surface (with iDCs expressing these receptors at a higher level than mDCs) whilst LLCs only expressed CD4 on their cell surface but had no detectable levels of CCR5 or CXCR4. As the goal was to eventually investigate the role of IL-27 in DCs with regard to HIV-1 infection, IL-27 treated DCs were also profiled and found to have similar levels of CD4, CCR5 and CXCR4 as iDCs or mDCs (see Figure 2A). Finally, the level of DC-SIGN, a C-type lectin important in promoting HIV-1 *trans* infection between DCs and T cells [31], was not differentially expressed between iDCs treated with or without IL-27 (Figure 2A). Next, each of the DC subtypes was infected with a CCR5 tropic virus, HIV-1_Ba-L_, or a CXCR4 tropic virus, HIV-1_NL4.3_ (Figure 2B). Not surprisingly, LLCs were unable to be infected with either HIV-1 virus presumably due to a lack of co-receptor expression of either CCR5 or CXCR4, providing an effective block at the entry level. iDCs were able to be infected with a CCR5 tropic virus but not a CXCR4 tropic virus. mDCs were able to be infected with either tropic virus but only at a low level – this may be due to the lower levels of co-receptor expression noted on flow cytometry which may have provided a partial block to entry of the virus into these cells. For future experiments, we chose to concentrate on HIV-1 infection predominantly on iDCs using the CCR5 tropic viral strain, HIV-1_Ba-L._ These findings are in keeping with the literature which has shown that iDCs are more susceptible to CCR5 tropic viruses compared to CXCR4 tropic viruses and that mature DCs are more likely to be infected by either CCR5 or CXCR4 tropic HIV-1 strains [32], [33].

### IL-27 Inhibits HIV-1 Infection in a Dose Dependent Manner in iDCs

**Figure 3:**
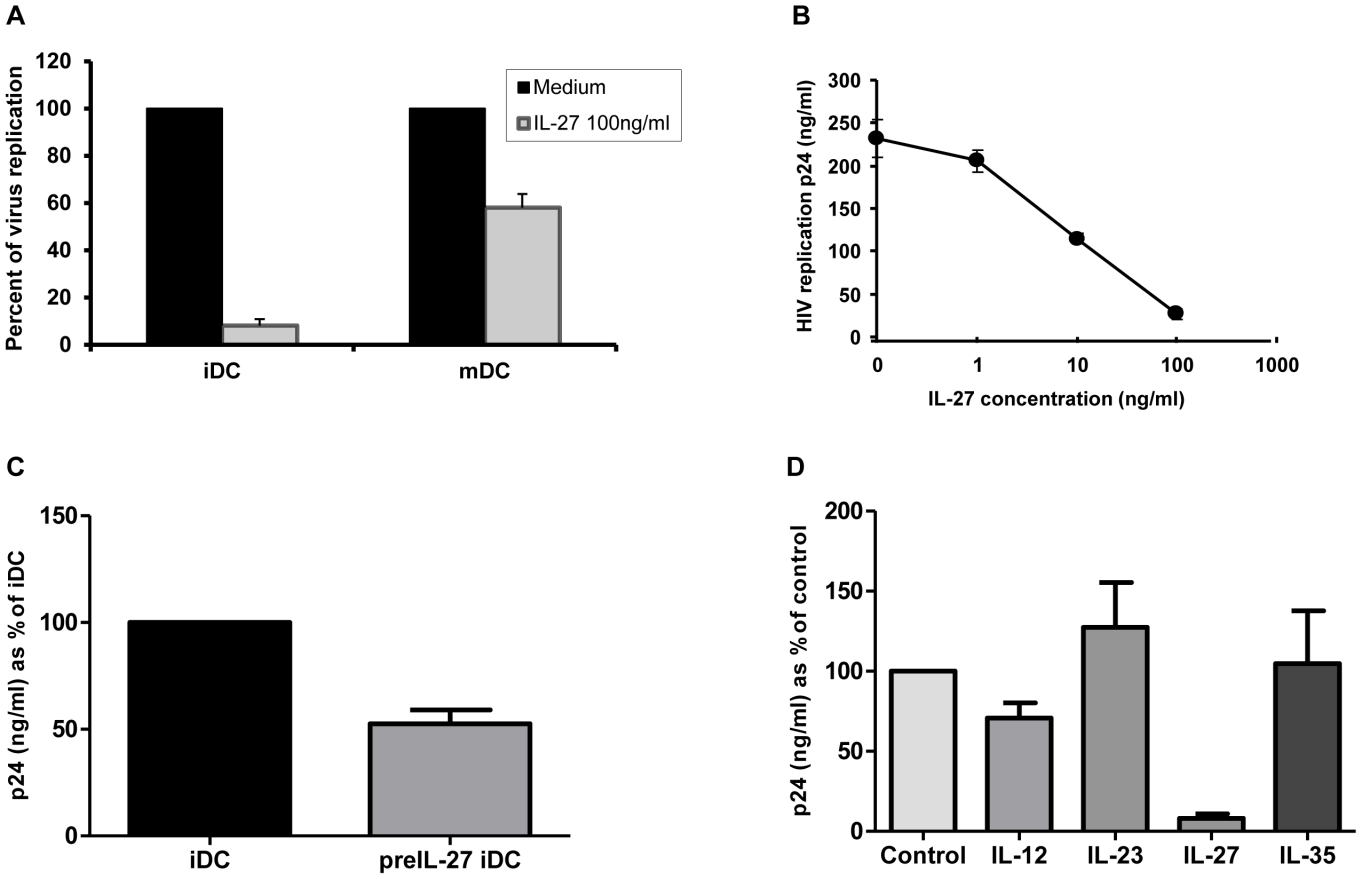
IL-27 inhibits HIV-1 replication in DCs in a dose-dependent manner. A: iDCs and mDCs were infected with HIV-1_Ba-L_, and HIV replication was measured by the HIV-1 p24 antigen capture kit. These experiments used DCs from three donors. B: HIV-infected iDCs were cultured in the presence of different amounts of IL-27 for 14 days and HIV-1 replication was monitored by the HIV-1 p24 antigen capture kit. C: iDCs were pre-treated with either media alone (iDCs) or 100 ng/ml IL-27 for 48 hours (PreIL-27-iDCs) followed by infection with HIV-1_Ba-L_. The HIV-1 infected iDCs were cultured for 14 days without IL-27 as described in the Materials and Methods and then HIV-1 replication was determined using the HIV-1 p24 antigen captures kit. The data shows a representative result from two independent donors. D: HIV-infected iDCs were cultured in the presence of 100 ng/ml of each of the IL-12 family of cytokines (IL-12, IL-23, IL-27 and IL-35) for 14 days and anti-viral effect of each cytokine was determined using the HIV p24 antigen capture kit. The results show the combined results of three independent donors and depict % of HIV-1 replication compared to untreated cells (control) and SE.

To investigate the role of IL-27 in HIV-1 infection of DCs, iDCs and mDCs were infected with the CCR5 tropic virus HIV-1_Ba-L,_ the cells were cultured in the presence or absence of 100 ng/ml of IL-27 and p24 levels were measured after 14 days to assess levels of *cis* HIV-1 infection (Figure 3A). IL-27 inhibited *cis* HIV-1 replication in iDCs and mDCs by 92±2.8% (n = 4) and 42±5.9% (n = 3), respectively. It should be noted that levels of infection were much higher in iDCs compared to mDCs (as per Figure 2B). To examine the optimal dose of IL-27 that could inhibit HIV-1 infection, a dose-response experiment was performed (Figure 3B). This demonstrated that IL-27, in a dose dependent manner, inhibited HIV-1 infection with ∼50% inhibition noted at a dose of 10 ng/ml and the maximal inhibition noted of approximately 90% at the highest dose used of 100 ng/ml. To further evaluate potential role of IL-27, HIV infection was performed on IL-27-pulse treated iDCs. In the study, following differentiation into iDCs, these cells were pre-treated with or without 100 ng/ml of IL-27 for 48 hours and then infected with HIV-1_Ba-L_. The infected cells were cultured in the absence of IL-27 for 14 days before p24 levels were checked. This showed that even pre-treatment of iDCs for 48 hours with IL-27 was able to decrease *cis* HIV-1 replication by 53±6.5% (n = 2) (Figure 3C). Finally, as IL-27 is part of the IL-12 family of cytokines, the other cytokines in this family (IL-12, IL-23 and IL-35) were assessed with regard to their possible role in inhibiting HIV-1 infection in DCs (Figure 3D). As compared to IL-27, none of the other IL-12 family members were able to significantly inhibit HIV-1 infection.

### IL-27 Induces a Post-entry Block to HIV-1 Infection

**Figure 4:**
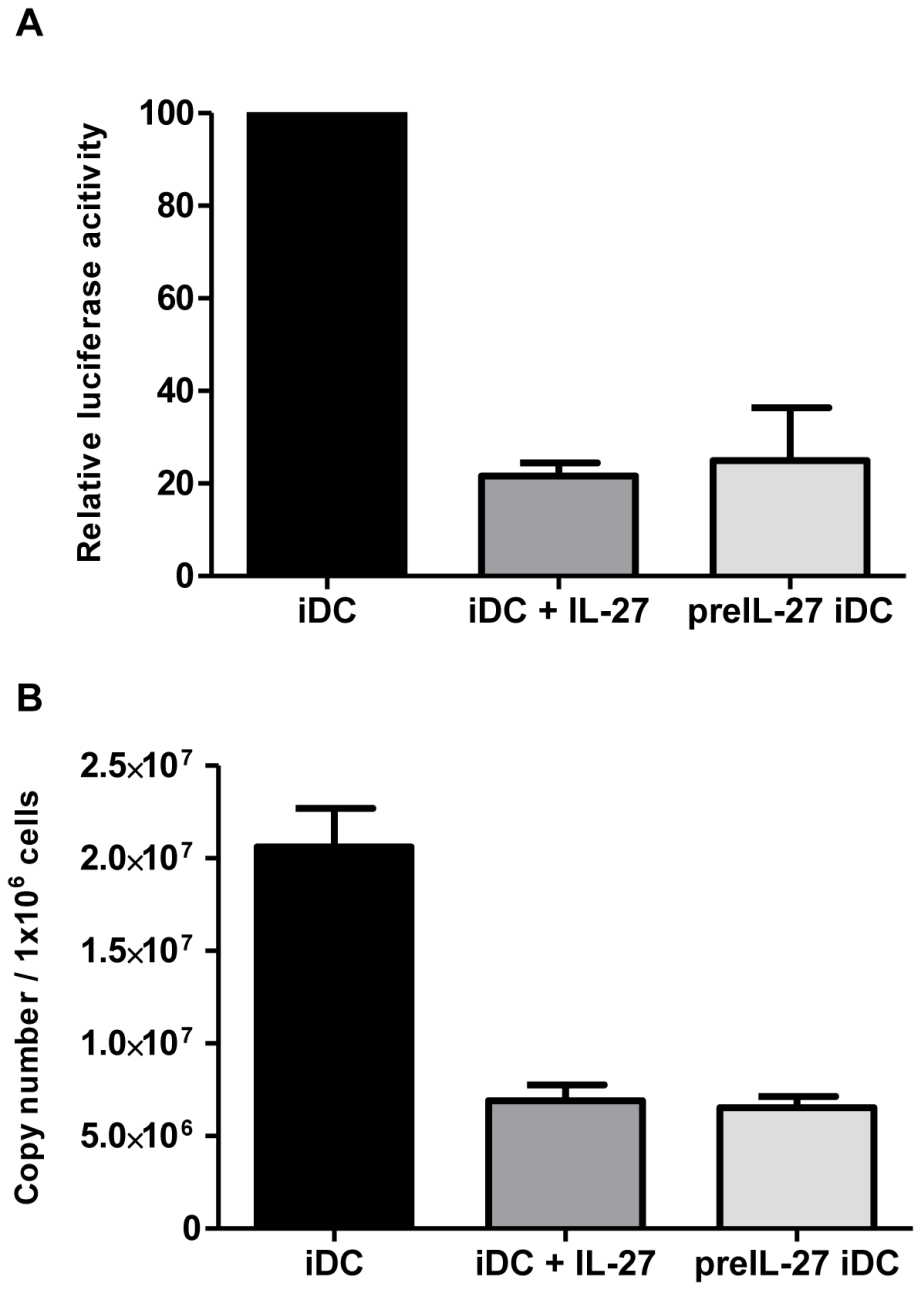
Inhibition of HIV-1 replication in DCs occurs after viral entry but before completion of reverse transcription. A: iDCs were pre-treated with IL-27 or mock treated for 48 hours. The treated cells were then infected with a HIV _NL4.3-Luc-VSVG_ virus followed by 4 days culture in the absence of IL-27 (Pre-IL-27 iDC). To compare the IL-27 effect, the mock-treated iDCs was also cultured in the presence of IL-27 (iDC+IL-27). Virus reporter activity was measured as described in the Materials and Methods B: The Late RT cDNA products were also semi quantitated using qPCR using the pseudotyped HIV infected cells. These experiments show the combined results of 4 separate donors. Error bars represent +/− SEM.

To try and understand at which level in the life cycle HIV-1 was being inhibited, iDCs were infected with a HIV-1- Luc-VSVG virus which theoretically would enable entry into all cells but would only be a single cycle infection due to a lack of HIV envelope gene in HIV genome. In iDCs treated with IL-27 or IL-27 pre-treated iDCs, there was a significant drop in luminescence observed as compared to mock treated iDCs infected with the pseudotyped virus (Figure 4A). This was suggestive that IL-27 treatment did not affect viral entry but its effect was occurring down-stream from this event. These results are consistent with the fact that IL-27 does not affect CD4 or co-receptor expression (Figure 2A). To look into this further, total RNA was extracted from iDCs infected with the pseudotyped virus and late RT cDNA products were measured using qRT-PCR (Figure 4B). In the IL-27 treated iDCs and IL-27 pretreated iDCs, late RT cDNA products were significantly reduced by 65±2.4% (n = 4) compared to untreated iDCs infected with HIV-1, suggesting that an inhibition was occurring post-entry and up to the level of reverse transcription.

### IL-27 Treatment of HIV-1 Infected DCs does not Up-regulate IFNs

**Figure 5:**
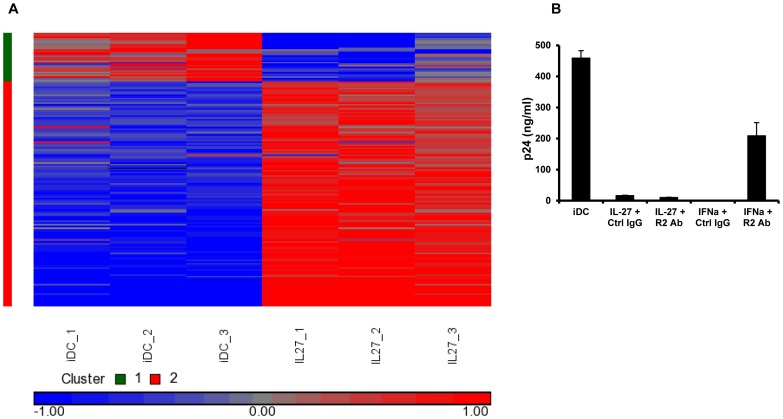
The anti-HIV effects of IL-27 on DCs are not mediated by Type I IFNs. A: iDCs from three independent donors were treated with or without IL-27 for 24 hours and then RNA was extracted. An Affymetrix microarray and gene expression profile analysis were performed as described in the Materials and Methods. B: HIV-infected iDCs were pretreated with anti-IFNαR antibody (R2Ab) or isotype control antibody (Ctrl Ab) before IL-27 treatment. As an assay control, the antibody-pretreated cells were also treated with IFN-α. This graph represents one donor from three independent donors. Error bars represent SD of replicates.

To try and understand the mechanism(s) whereby IL-27 is inhibiting HIV-1 in DCs, a gene expression microarray of IL-27 treated iDCs was performed (see Figure 5A). Compared to mock treated iDCs, this revealed 158 genes which were differentially expressed by IL-27 treatment (up- or down-regulated more than 2 fold, corrected p value of <0.05, upregulated genes are listed in Table S1 and downregulated genes in Table S2). Of these genes, 129 were up-regulated in IL-27 treated iDCs compared to mock treated. Some of the up-regulated genes were IFN-inducible genes, including the anti-viral genes, MX1 (5.4 fold) and OAS2 (3.8 fold) in IL-27 treated iDCs. A total of 29 genes were down-regulated in the IL-27 treated iDCs. Recently, several host restriction factors against HIV-1 have been described including APOBEC3G, APOBEC3F, BST2 and SAMHD1. The microarray analysis did not demonstrate any changes in any of these well described host restriction factors that may have explained the anti-HIV phenotype seen in IL-27 treated iDCs.

Interferons, on the other hand, are potent antiviral cytokines which may provide a rational explanation for the anti-viral phenotype noted with IL-27. Of note, the microarray showed no differential expression of Type I IFNs in IL-27 treated iDCs compared to mock treated iDCs. To investigate the potential role of Type I IFNs in our system, RNA was extracted at various time points (0, 6, 12, 24 and 48 hours) after IL-27 was added to iDCs and qRT-PCR was used to measure IFN-α and IFN-β at the mRNA level. This showed no significant change in expression of Type I IFNs in IL-27 treated iDCs compared to untreated iDCs (data not shown). To investigate in more depth whether or not the IFNs were contributing to the anti-HIV effects of IL-27, the supernatants of IL-27 treated iDCs were collected at 48 hours exposure post IL-27 and screened for IFN-α or IFN-β at the protein level. No detectable Type I IFN was detected in either the IL-27 treated or mock treated iDCs (data not shown). Finally, to definitively exclude IFN as mediators of anti-viral defense in IL-27 treated iDCs, IL-27 was able to effectively inhibit HIV-1 in iDCs in the presence of a neutralizing antibody to type-I IFN receptors (Figure 5B). In a positive control assay for the neutralization, administration of the IFN-α blocking antibody partially restored the ability of anti-HIV in the presence of 10 U/ml of IFNα in iDCs. Overall these data indicated that IFNs are not involved in the IL-27 inhibition of HIV replication in iDCs. For completeness, chemokines that could bind to CCR5, including RANTES, MIP1α and MIP1β, were screened for and no significant difference was noted in chemokine expression between IL-27 treated and untreated iDCs using qRT-PCR and ELISA (data not shown).

## Discussion

In this study, DC subsets expressed high levels of the IL-27Rα subunit and were shown to respond to IL-27 by phosphorylating STATs -1, -3 and -5 rapidly after treatment with IL-27. IL-27, in a dose-dependent manner, potently inhibited the CCR5 tropic virus, HIV-1_Ba-L_ in both iDCs and mDCs while other IL-12 family members (IL-12, IL-23 and IL-35) had no effect on HIV-1 viral replication. Type I IFNs played no role in the antiviral effects of IL-27 treatment of DCs. Further mechanistic studies showed that the block in the HIV-1 pathway existed between virus entry and prior to completion of reverse transcription, although further work is required to find the exact mechanism of inhibition.

This study is the first report showing that IL-27 has significant inhibitory effects on *cis* HIV-1 replication in DCs. It adds to other studies that demonstrate IL-27 can inhibit HIV-1 replication in CD4+ T cells [8] and macrophages [8], [9], [10]. In those studies, IL-27 appears to have a stronger inhibitory action in macrophages compared to CD4+ T cells and this may be related to signaling differences between these different cells which may subsequently affect the up-regulation of different host restriction factors effective against HIV-1. Similar to prior reports, type I IFNs were not found to be important in mediating the antiviral effects seen [10], although some interferon inducible genes, such as MX1 and OAS2 were detected on the microarray of IL-27 treated DCs. We have demonstrated that IL-27 induces the activation of various STAT proteins (STATs-1, -3 and -5 but not STAT-2) whilst IFN-α is a potent stimulator of phosphorylation of all STAT proteins. It is therefore not surprising that some IFN-inducible genes are also up-regulated with IL-27 treatment as common STAT pathways are also stimulated as we previously described in macrophages [10]. In our manuscript, we have convincingly demonstrated that despite the induction of IFN related genes, the anti-HIV effect of IL-27 is not mediated via the actions of IFN (see Figure 5B). Previously Type I IFN has been reported to act as an intermediary in the antiviral action of IL-27 in monocyte derived macrophages [9]. However, the use of different culture media between groups to differentiate monocytes to macrophages may explain why IFNs were excluded as being major players in mediating the anti-HIV-1 effects of IL-27 in macrophages [8], [10]. In this study, the lack of detectable IFNα or IFNβ in the supernatant of IL-27 treated iDCs and the fact that an IFN neutralizing antibody when added to IL-27 treated iDCs did not abrogate the antiviral effect, strongly suggest that IFN is not acting as an intermediary protective factor post IL-27 treatment. Interestingly, pre-treatment of iDCs with IL-27 for just 48 hours, seemed to have a similar protective effect as culturing HIV-1 infected cells with IL-27 for 14 days. This is suggestive that changes that result in a protective phenotype occur early after IL-27 signaling and these changes persist during the life course of the cell. As the infection of a pseudo type HIV-1 virus, which could only result in a single round of infection, was also inhibited by IL-27 treatment and the proviral copy number was decreased in the treatment arm, this suggested that IL-27 works by suppressing HIV-1 at a certain step in the HIV life cycle. This assay system, however, cannot exclude that other down-stream processes in the HIV-1 replication pathway are also not affected by IL-27 but at least one significant block is occurring post-viral entry and prior to the completion of reverse transcription. The expression level of IL-27Rα (WSX-1) on three different DC subtypes (iDCs, mDCs and LLCs) was also examined. Monocytes were differentiated into iDCs by GM-CSF and IL-4, and mDCs were induced from iDC using LPS. The expression level of IL-27Rα on iDCs was at comparative levels to that on monocytes, indicating that the differentiation process using a combination of GM-CSF and IL-4 (in G4 media) has no impact *per se* on IL27Rα expression. Intriguingly, the expression of IL-27Rα was down-regulated on LLCs. LLCs were induced from monocytes in the presence of G4 media with TGF-β, suggesting that the TGF-β may interfere with IL-27 signaling by preventing IL27Rα expression. It is reported that IL-27 suppresses development of regulatory T cells (Tregs) [34], whilst Tregs require TGF-β and IL-2 to differentiate from a Th0 cell type [35]. Further study is needed to understand the interplay between IL-27 and TGF-β from a functional perspective.

There have been two reported studies of the levels of IL-27 in plasma in patients with HIV-1 infection. The first study, which had a total of 53 patients, showed a weak negative correlation between IL-27 plasma levels and HIV-1 viral load [36], suggestive that the virus down-regulates IL-27 for its own advantage (as IL-27 has anti-HIV properties). A much larger study with 120 HIV-1 positive patients and 108 HIV-1 uninfected controls, found that IL-27 levels were significantly higher in HIV-1 infected patients compared to uninfected controls (p<0.001) and a small positive correlation was noted between IL-27 levels with CD4+ T cell count, in keeping with down-regulation of IL-27 with increasing immunosuppression (decreased CD4+ T cell count) [37]. Conclusions regarding plasma IL-27 levels are difficult to formulate as the significant effect of IL-27 is likely to be occurring in local areas such as in lymphoid tissue and the overall levels of IL-27 (as measured in plasma) may not reflect biologically relevant levels in tissues of interest. One way in which IL-27 may play a role in HIV-1 pathogenesis is in the increased levels of IL-10 noted in HIV-1 infection [38]. Recently, IL-27 has also been shown to amplify IL-10 secretion in cytotoxic T lymphocytes through a Blimp-1 dependent mechanism [39]. The increased IL-27 noted in the plasma of patients with HIV-1 may be one reason why IL-10 levels have also been noted to be higher in HIV-1 infected patients.

DCs have important roles in HIV-1 infection in terms of disseminating the virus to CD4+T cells but also in initiating adaptive immune responses against the virus. DCs can be infected with HIV-1 in two distinct ways: *trans*-infection can occur via infectious synapses or via exocytosis of HIV-associated exosomes [14] and *cis*-infection following de novo viral production in DCs [15]. The findings presented suggest that IL-27 is able to suppress *cis-*infection. Previous work has shown that IL-27 inhibits HIV replication in T cells [8] and IL-27 suppresses R5 HIV infection in CD4+ T cells (unpublished data, T. Imamichi et al.), thus it is reasonable to predict that IL-27 will also able to inhibit *trans*-infection from DC to CD4+T cells. If this were the case, the inhibition of *trans* HIV-1 would not be due to differences in DC-SIGN expression on DCs which were shown not to be affected by IL-27 treatment. Now that IL-27 has shown potent anti-HIV properties in three important cell types in HIV-1 infection (CD4+ T cell, macrophage and DC), the question remains whether this cytokine may be able to be utilized in the clinical setting. By our calculations, if we assume a ∼65% reduction of HIV-1 replication with each cycle of HIV-1 infection with IL-27 treatment and an end result of ∼90% reduction in p24 after 14 days of treatment, we estimate that approximately three cycles of HIV-1 infection have taken place. The assumption made is that the wild type virus behaves similarly to the VSVG pseudotyped virus which may not necessarily be the case. This suggests that IL-27 is a potent anti-HIV-1 agent and that IL-27 therapy may therefore find utility in primary HIV-1 infection where highly potent treatments are predicted to be particularly useful. As IL-27 can significantly reduce HIV-1 infection in DCs, a vitally important cell type early in infection, one may speculate whether this could be used in a prophylactic sense or in combination with a topical microbicide such as tenofovir gel to reduce the chance of acquiring HIV-1 or reducing the viral set-point. There is an increasingly strong case to see whether this cytokine may have therapeutic benefits. One would imagine that animal models of infection (using SIV in rhesus macaques for example) will be required to test the efficacy of IL-27 prior to Phase I clinical trials beginning. In summary, in this study, we have demonstrated that: (1) iDC and mDC but not LLC expresses the IL-27 receptor on the cell surface (2) IL-27 treatment enhances the expression of some DC maturation markers (CD11c, CD86, HLA-ABC and HLA-DR) similar to LPS-treated iDCs (3) IL-27 induces activation of STAT-1,-3, -5 in iDCs (4) IL-27, but not other IL-12 family cytokines (IL-12, IL-23, IL-35) inhibits HIV in iDCs without intermediate IFNs. Although it is clear that IL-27 does not mediate its effect through IFN in DCs, more work is required to fully understand the mechanism(s) of how IL-27 mediates anti-HIV effects in multiple cell types, including DCs.

## Supporting Information

Figure S1 **Maturation markers on DCs treated with either IL-27 or LPS.** iDCs were treated with either 100 ng/ml IL-27 or 1 µg/ml LPS and DC maturation markers were measured by flow cytometry as described in the Materials and Methods. In the plots, green lines represent the isotype control, blue lines represent mock treated cells and red lines represent either IL-27 treated or LPS treated cells as marked on the figure.
(TIF)

Table S1 Upregulated genes in IL-27 treated iDCs. iDCs from three independent donors were stimulated with or without IL-27 for 48 hours, and then the gene expression profile was analyzed using the human GeneArray ST 1.0 microarray (Affymetrix) as discrived in the Material and Methods. The table shows a list of up-regulated genes in IL-27–treated cells, fold change compared to untreated cells and p value of each gene.
(DOCX)

Table S2 Downregulated genes in IL-27 treated iDCs. iDCs from three independent donors were stimulated with or without IL-27 for 48 hours, and then the gene expression profile was analyzed using the human GeneArray ST 1.0 microarray (Affymetrix) as discrived in the Material and Methods. The table shows a list of down-regulated genes in IL-27–treated cells, fold change compared to untreated cells and p value of each gene.
(DOCX)
