# Supplementary figures and images for: Interleukin-27 Is a Potent Inhibitor of cis HIV-1 Replication in Monocyte-Derived Dendritic Cells via a Type I Interferon-Independent Pathway
Source: PLoS One. 2013 Mar 20;8(3):e59194. doi: 10.1371/journal.pone.0059194 (PMC3604098; doi:10.1371/journal.pone.0059194)

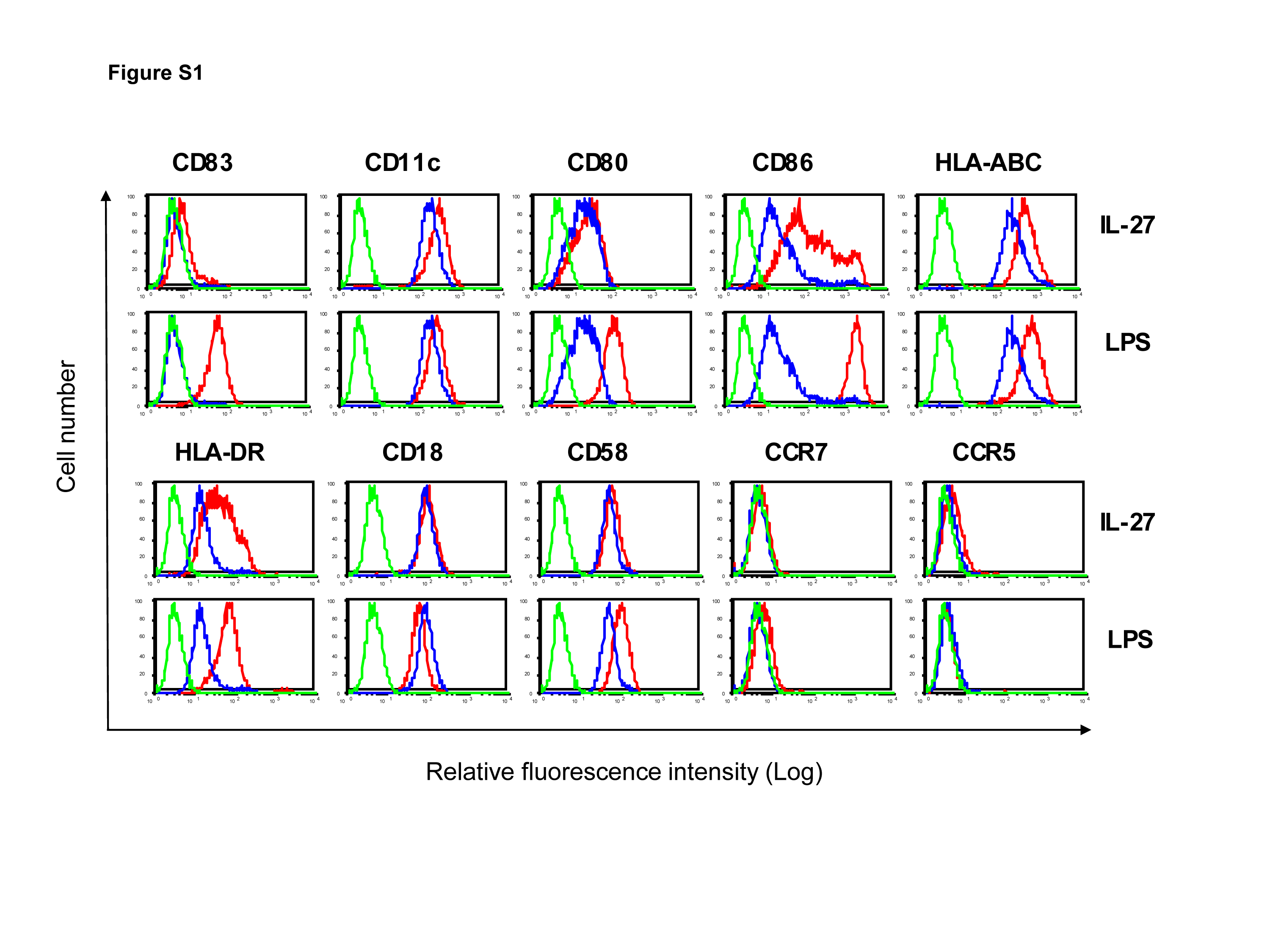

Supplement: Figure S1 — Maturation markers on DCs treated with either IL-27 or LPS. iDCs were treated with either 100 ng/ml IL-27 or 1 µg/ml LPS and DC maturation markers were measured by flow cytometry as described in the Materials and Methods. In the plots, green lines represent the isotype control, blue lines represent mock treated cells and red lines represent either IL-27 treated or LPS treated cells as marked on the figure. (TIF) [file pone.0059194.s001.tif]
