# Supplementary material for: Interleukin-27 Is a Potent Inhibitor of cis HIV-1 Replication in Monocyte-Derived Dendritic Cells via a Type I Interferon-Independent Pathway
Source: PLoS One. 2013 Mar 20;8(3):e59194. doi: 10.1371/journal.pone.0059194 (PMC3604098; doi:10.1371/journal.pone.0059194)
Supplement: Table S1 — Upregulated genes in IL-27 treated iDCs. iDCs from three independent donors were stimulated with or without IL-27 for 48 hours, and then the gene expression profile was analyzed using the human GeneArray ST 1.0 microarray (Affymetrix) as discrived in the Material and Methods. The table shows a list of up-regulated genes in IL-27–treated cells, fold change compared to untreated cells and p value of each gene. (DOCX) [file pone.0059194.s002.docx]

**Table S1: Upregulated genes in IL-27 treated iDCs**

| **Gene name** | **Fold change** | **p value** |
| --- | --- | --- |
| CXCL9 | 46.6 | 0.0155 |
| SERPING1 | 46.2 | 0.0022 |
| IFI44L | 32.5 | 0.0036 |
| GBP4 | 25.5 | 0.0068 |
| ANKRD22 | 24.2 | 0.0187 |
| RARRES3 | 14.2 | 0.0046 |
| GBP5 | 13.5 | 0.0011 |
| APOBEC3A | 12.0 | 0.0205 |
| IFIT3 | 9.7 | 0.0203 |
| GBP1 | 9.7 | 0.0124 |
| TNFSF10 | 9.1 | 0.0100 |
| GCH1 | 8.9 | 0.0335 |
| IFIT2 | 8.4 | 0.0203 |
| CCL8 | 8.4 | 0.0382 |
| IFI27 | 7.4 | 0.0040 |
| IFITM1 | 7.1 | 0.0016 |
| IRF1 | 6.0 | 0.0081 |
| MX1 | 5.4 | 0.0306 |
| IFITM3 | 5.3 | 0.0488 |
| SLAMF7 | 5.2 | 0.0339 |
| FAM26F | 4.6 | 0.0184 |
| EPSTI1 | 4.5 | 0.0045 |
| HAPLN3 | 4.5 | 0.0167 |
| LGALS3BP | 4.5 | 0.0155 |
| OAS3 | 4.5 | 0.0192 |
| FRMD3 | 4.4 | 0.0366 |
| SNX10 | 4.4 | 0.0447 |
| GBP2 | 4.4 | 0.0036 |
| AIM2 | 4.3 | 0.0232 |
| GIMAP6 | 4.2 | 0.0032 |
| APOL1 | 4.2 | 0.0124 |
| DDX60 | 4.1 | 0.0032 |
| LDLR | 4.1 | 0.0186 |
| CTLA4 | 4.1 | 0.0133 |
| GRIN3A | 4.1 | 0.0117 |
| STAT1 | 4.0 | 0.0146 |
| BATF2 | 3.9 | 0.0204 |
| OAS2 | 3.8 | 0.0022 |
| GIMAP8 | 3.8 | 0.0312 |
| WARS | 3.8 | 0.0124 |
| RSAD2 | 3.8 | 0.0173 |
| P2RY12 | 3.7 | 0.0124 |
| GIMAP4 | 3.7 | 0.0007 |
| TAP1 | 3.6 | 0.0078 |
| IFI6 | 3.5 | 0.0060 |
| PARP9 | 3.5 | 0.0023 |
| ALDH1A1 | 3.5 | 0.0195 |
| VAMP5 | 3.5 | 0.0088 |
| CYBB | 3.5 | 0.0180 |
| C11orf75 | 3.5 | 0.0061 |
| IFI44 | 3.4 | 0.0083 |
| PSMB9 | 3.4 | 0.0030 |
| IFIT1 | 3.3 | 0.0049 |
| NLRC5 | 3.2 | 0.0070 |
| GIMAP5 | 3.2 | 0.0017 |
| MYOF | 3.1 | 0.0092 |
| PSME2 | 3.1 | 0.0062 |
| SLC8A1 | 3.1 | 0.0080 |
| USP18 | 3.0 | 0.0461 |
| GVINP1 | 3.0 | 0.0001 |
| FPR2 | 3.0 | 0.0343 |
| RHBDF2 | 3.0 | 0.0279 |
| TAP2 | 3.0 | 0.0060 |
| PIM1 | 3.0 | 0.0369 |
| SLC6A12 | 3.0 | 0.0105 |
| IL31RA | 2.9 | 0.0292 |
| BTN3A2 | 2.8 | 0.0007 |
| CFB | 2.8 | 0.0094 |
| APOL6 | 2.8 | 0.0003 |
| LAP3 | 2.7 | 0.0012 |
| IFI35 | 2.7 | 0.0100 |
| SECTM1 | 2.6 | 0.0019 |
| XAF1 | 2.5 | 0.0031 |
| GBP3 | 2.5 | 0.0009 |
| BTN3A3 | 2.5 | 0.0088 |
| PARP14 | 2.5 | 0.0058 |
| IFIH1 | 2.5 | 0.0171 |
| SLC44A2 | 2.5 | 0.0008 |
| USP6NL | 2.5 | 0.0405 |
| GIMAP7 | 2.4 | 0.0312 |
| CYSLTR2 | 2.4 | 0.0170 |
| TMEM140 | 2.4 | 0.0157 |
| UBE2L6 | 2.3 | 0.0028 |
| CASP1 | 2.3 | 0.0034 |
| CMPK2 | 2.3 | 0.0367 |
| ERAP2 | 2.3 | 0.0184 |
| ETV7 | 2.3 | 0.0499 |
| FYB | 2.3 | 0.0026 |
| IRF8 | 2.3 | 0.0365 |
| ODF3B | 2.3 | 0.0195 |
| IFIT5 | 2.3 | 0.0177 |
| HERC5 | 2.3 | 0.0014 |
| SP110 | 2.2 | 0.0174 |
| IL6 | 2.2 | 0.0136 |
| LY6E | 2.2 | 0.0025 |
| APOL2 | 2.2 | 0.0022 |
| DDX58 | 2.2 | 0.0169 |
| NMI | 2.2 | 0.0075 |
| TLR1 | 2.2 | 0.0181 |
| SP140 | 2.2 | 0.0303 |
| RNF213 | 2.2 | 0.0008 |
| STAT2 | 2.2 | 0.0008 |
| CFH | 2.2 | 0.0004 |
| RNF213 | 2.1 | 0.0027 |
| C4A | 2.1 | 0.0247 |
| ALPK1 | 2.1 | 0.0042 |
| ASPHD2 | 2.1 | 0.0226 |
| STOM | 2.1 | 0.0294 |
| TRPM2 | 2.1 | 0.0283 |
| XRN1 | 2.1 | 0.0203 |
| RCN1 | 2.1 | 0.0396 |
| C1QA | 2.1 | 0.0092 |
| OAS1 | 2.1 | 0.0412 |
| SLC31A2 | 2.1 | 0.0172 |
| HCP5 | 2.1 | 0.0219 |
| FAM20A | 2.1 | 0.0455 |
| GIMAP2 | 2.1 | 0.0063 |
| TRIM22 | 2.1 | 0.0100 |
| TRANK1 | 2.1 | 0.0304 |
| RTP4 | 2.1 | 0.0098 |
| N4BP2L1 | 2.0 | 0.0328 |
| CASP7 | 2.0 | 0.0095 |
| DTX3L | 2.0 | 0.0005 |
| TYMP | 2.0 | 0.0148 |
| APOBEC3D | 2.0 | 0.0349 |
| TRAFD1 | 2.0 | 0.0035 |
| FPR1 | 2.0 | 0.0027 |
| SDC3 | 2.0 | 0.0141 |
| FGD6 | 2.0 | 0.0239 |
