# Supplementary material for: Interleukin-27 Is a Potent Inhibitor of cis HIV-1 Replication in Monocyte-Derived Dendritic Cells via a Type I Interferon-Independent Pathway
Source: PLoS One. 2013 Mar 20;8(3):e59194. doi: 10.1371/journal.pone.0059194 (PMC3604098; doi:10.1371/journal.pone.0059194)
Supplement: Table S2 — Downregulated genes in IL-27 treated iDCs. iDCs from three independent donors were stimulated with or without IL-27 for 48 hours, and then the gene expression profile was analyzed using the human GeneArray ST 1.0 microarray (Affymetrix) as discrived in the Material and Methods. The table shows a list of down-regulated genes in IL-27–treated cells, fold change compared to untreated cells and p value of each gene. (DOCX) [file pone.0059194.s003.docx]

**Table S2: Downregulated genes in IL-27 treated iDCs**

| **Gene name** | **Fold change** | **p value** |
| --- | --- | --- |
| DHRS2 | 4.57 | 0.0437 |
| ADAM23 | 4.31 | 0.0274 |
| OR10J3 | 4.05 | 0.0198 |
| DUOX1 | 3.38 | 0.0434 |
| PLEKHA5 | 3.37 | 0.0474 |
| GOLGA8B | 2.92 | 0.0351 |
| GOLGA8B | 2.91 | 0.0303 |
| BCL11A | 2.85 | 0.0316 |
| WDFY4 | 2.71 | 0.0198 |
| ADAM19 | 2.65 | 0.0024 |
| DNASE1L3 | 2.49 | 0.0477 |
| CALCRL | 2.44 | 0.0279 |
| GNG2 | 2.41 | 0.0024 |
| SNORD12C | 2.40 | 0.0134 |
| CXCR1 | 2.39 | 0.0139 |
| HSPA2 | 2.37 | 0.0295 |
| GCOM1 | 2.35 | 0.0321 |
| WDFY4 | 2.31 | 0.0220 |
| ITM2C | 2.28 | 0.0031 |
| ICAM3 | 2.27 | 0.0062 |
| NET1 | 2.16 | 0.0094 |
| KLF8 | 2.14 | 0.0037 |
| SRGAP1 | 2.11 | 0.0204 |
| HSPC159 | 2.11 | 0.0299 |
| DUOXA1 | 2.10 | 0.0339 |
| SNORD104 | 2.06 | 0.0206 |
| KLF12 | 2.05 | 0.0436 |
| XKR3 | 2.03 | 0.0291 |
| PGA3 | 2.01 | 0.0335 |
| LOC653075 | 2.01 | 0.0471 |
